# Phase I trial combining sulfasalazine and gamma knife radiosurgery for recurrent glioblastoma^☆^

**DOI:** 10.1016/j.mex.2026.103915

**Published:** 2026-04-22

**Authors:** Bente Sandvei Skeie, Linda Sleire, Eli Renate Grüner, Sidsel Bragstad, Jan Ingeman Heggdal, Geir Egil Eide, Per Øyvind Enger

**Affiliations:** a Department of Neurosurgery, Haukeland University Hospital (HUS), Jonas Lies vei 65, Bergen, 5021, Norway; b Department of Biomedicine, University of Bergen (UiB), Jonas Lies vei 91, Bergen, 5021, Norway; c Laboratory Medicine and Pathology, HUS, Jonas Lies vei 65, Bergen, 5021, Norway; d Department of Physics and Technology, UiB, Jonas Lies vei 65, Bergen, 5021, Norway; e Mohn Medical Imaging and Visualization Center, Department of Radiology, HUS, Jonas Lies vei 65, Bergen, 5021, Norway; f Department of Oncology and Medical Physics, HUS, Jonas Lies vei 65, Bergen, 5021, Norway; g Department of Neurosurgery, Stavanger University Hospital, Armauer Hansens vei 30, Stavanger, 4011, Norway

**Keywords:** Sulfasalazine, High grade glioma, Glioblastoma, Recurrence, Stereotactic radiosurgery, Gamma knife radiosurgery, Radiosensitizer

## Abstract

Recurrent glioblastoma (GBM) remains a therapeutic challenge with poor prognosis and limited treatment options. This phase I, open-label, dose-escalation trial investigates the safety and tolerability of combining sulfasalazine, a cystine/glutamate antiporter-inhibitor, with gamma knife radiosurgery (GKRS) for patients with recurrent GBM. Sulfasalazine is hypothesized to potentiate radiotherapy by depleting tumor cells of glutathione (GSH), thereby enhancing susceptibility to reactive oxygen species generated during irradiation. The study aims to establish the maximum tolerated dose (MTD) of sulfasalazine in combination with GKRS and explore preliminary efficacy signals through imaging and clinical endpoints.

• Primary objective: Determine the MTD of sulfasalazine combined with GKRS and recommend a dose for future
• phase II trials using the common toxicity criteria for adverse events v4.0.
• Secondary objectives: Assess changes in intratumoral GSH via GSH‑edited Magnetic Resonance Spectroscopy, tumor response by Response Assessment in Neuro‑Oncology criteria, metabolic response on Carbon-11 methionine positron emission tomography, quality of life, progression free and overall survival.
• Design: Open-label, dose-escalation study enrolling 12–24 patients with recurrent GBM eligible for GKRS.

Patients receive sulfasalazine for 3 days prior to GKRS, followed by 12 months of monitoring for adverse events, imaging biomarkers, radiological and survival outcomes. This trial will provide safety data and inform the potential of sulfasalazine as a radiosensitizer in GBM treatment.

## Graphical abstract

**Figure:**
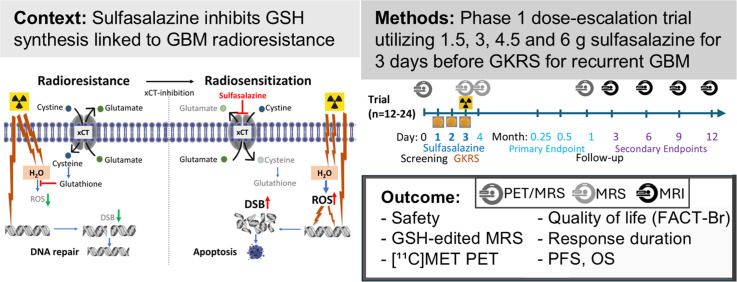

## Specifications table

**Table:** 

Subject area	Medicine and Dentistry
**More specific subject area**	Radiosensitization for glioblastoma recurrence
**Name of your protocol**	Phase I trial combining sulfasalazine and gamma knife radiosurgery for recurrent glioblastoma
**Reagents/tools**	Sulfasalazine EN «Pfizer», Gamma knife (Elekta Icon)
**Experimental design**	Open label phase I 3 + 3 dose escalating design
**Trial registration**	ClinicalTrials.gov Identifier: NCT04205357
**Ethics**	The trial was approved by the regional ethical committee (REK vest 6834) and the European Medicines Agency (EUDRACT No.2019–002,110-40). Informed consent was obtained from all trial participants.
**Value of the Protocol**	• Introduces a novel radiosensitization strategy by targeting tumor redox homeostasis through cystine/glutamate antiporter system x_C_^−^ inhibition, addressing a key radioresistance mechanism in glioblastoma. • Provides first-in-human safety and tolerability data for sulfasalazine combined with stereotactic radiosurgery, alongside patient reported quality of life assessments. • Offers mechanistic evidence of treatment effect through glutathione‑edited Magnetic Resonance Spectroscopy) and preliminary efficacy data via Carbon-11 methionine positron emission tomography and Magnetic Resonance imaging.

## Background

Glioblastoma (GBM) is the most common malignant primary brain tumor in adults, with an incidence of 3–4 per 100,000 and a median age at diagnosis of 65 years[1]. Despite multimodal treatment with surgical resection, high dose radiotherapy, and temozolomide, GBM remains incurable, and recurrence is inevitable[2]. At recurrence the therapeutic options are limited, and there is no established standard of care. Management is largely palliative, balancing potential benefits with quality of life (QoL).

Current strategies for recurrent GBM include re-resection, systemic chemotherapy, bevacizumab, tumor treating fields and conventional re-irradiation or stereotactic radiosurgery (SRS). *Re*-resection is feasible in only 20% of cases and carries significant morbidity[3]. Resistance to temozolomide develops rapidly. Lomustine[4], bevacizumab[5], and tumor treating fields[6] have shown limited efficacy. Although conventional fractionated radiotherapy is the most effective part of the initial treatment[7], it is rarely repeated at recurrence due to the risk of neurocognitive side effects, making SRS an attractive alternative. SRS delivers single session high-dose, focal radiation to recurrent tumors while sparing surrounding brain tissue, offering a favorable safety profile and efficacy compared to surgery[8], chemotherapy and conventional radiotherapy[9]. Still, the therapeutic impact of SRS is constrained by GBM radioresistance and prior high‑dose radiation exposure. In a retrospective study using an 18 Gy prescription dose, almost half of the patients ultimately required surgical resection due to radiation necrosis[10], highlighting the need for improved radiation strategies.

Radioresistance is in part mediated by antioxidant defenses such as glutathione (GSH)[11]. Tumor cells neutralize radiation-induced reactive oxygen species (ROS), preventing DNA damage and cell death. The cystine/glutamate antiporter system x_C_^−^ (xCT), providing cysteine uptake needed for GSH synthesis[12] is upregulated in several types of cancers[13]. Sulfasalazine, an FDA-approved anti-inflammatory drug, inhibits xCTr[12]. We have previously shown that xCT was minimally expressed in healthy brain tissue and markedly upregulated in 30 GBM biopsies from our institutional tumor bank[14]. Moreover, our preclinical studies demonstrated that sulfasalazine depletes GSH, increases ROS, and synergistically enhances radiation-induced DNA damage in glioma cell lines and significantly prolong survival in a GBM xenograft models when combined with gamma knife radiosurgery (GKRS) compared to either treatment alone[14]. This protocol describes the first-in-human Phase I trial evaluating sulfasalazine as a radiosensitizer in combination with GKRS for recurrent GBM. The study aims to establish the maximum tolerated dose of sulfasalazine, the recommended dose for a future phase II trial and to assess the preliminary efficacy through advanced imaging (GSH‑edited Magnetic Resonance Spectroscopy (GSH‑edited MRS)for GSH depletion, Carbon-11 methionine positron emission tomography ([¹¹C]methionine PET) for metabolic tumor response) as well as clinical outcomes including QoL and survival.

## Description of protocol

### Study rationale

Recurrent glioblastoma carries a dismal prognosis and lacks effective treatment options, creating a clear need for innovative therapeutic strategies. Recurrence typically develops locally at the resection margin, making focal retreatment an attractive approach, however, the effect of GKRS is constrained by prior irradiation and the inherent radioresistance of glioblastoma. Preclinical studies show that short‑term sulfasalazine exposure is sufficient to selectively deplete intratumoral GSH, inhibiting xCT‑mediated cystine uptake and thereby increasing susceptibility to radiation‑induced oxidative stress, prolonging survival without toxicity in experimental models. This selective radiosensitizing effect enhances the radiation response in tumor tissue while sparing normal brain supporting clinical evaluation of sulfasalazine repurposed as a radiosensitizer.

Sulfasalazine has a well-known safety profile with mostly mild gastrointestinal adverse events. However, GBM patients often take multiple concomitant medications and have compromised health status, which could increase their susceptibility to adverse effects. Earlier glioma trials [15,16] using prolonged sulfasalazine dosing reported substantial neurological toxicity in patients with large recurrences and low performance status, or hematologic toxicity when sulfasalazine was administered long‑term together with temozolomide, indicating that a brief sulfasalazine regimen is essential to avoid cumulative drug toxicity. This phase I study therefore evaluates a three‑day sulfasalazine course prior to GKRS using clinically familiar sulfasalazine dosing, a conservative GKRS prescription dose [8], and strict eligibility criteria to minimize patient risk while assessing safety. If the combination is well tolerated and shows early indications of activity, these findings would justify progression to a phase II trial and further development of this treatment approach for recurrent glioblastoma.

### Aims of study

The primary aim of this study is to evaluate the safety of sulfasalazine in combination with GKRS determining the maximum tolerated dose and recommended phase II dose. The secondary aim is to explore preliminary anti‑tumor efficacy, including changes in intratumoral glutathione measured by GSH‑edited MRS; Magnetic Resonance Imaging (MRI)‑based tumor response at planned intervals; progression‑free (PFS) and overall survival (OS); [¹¹C]methionine PET ‑based assessment of metabolic response; patient‑reported QoL; functional status by Karnofsky Performance Status (KPS); corticosteroid use; and associations between prognostic markers and treatment response.

### Study design

This is a phase I, single‑center dose‑escalation trial using a classic 3 + 3 design [17] to evaluate escalating doses of oral sulfasalazine administered for three days prior to GKRS with an anticipated recruitment period of 24 months. The dose will be escalated or de-escalated with diminishing fractions of the preceding dose (modified Fibonacci sequence), depending on the occurrence of adverse events grade ≥ 3.

Cohorts of three patients receive escalating once‑daily sulfasalazine doses (1.5, 3, 4.5, 6 g) for three days, with GKRS on Day 3. Escalation proceeds if no ≥ grade 3 toxicity occurs; a single ≥ grade 3 event expands the cohort to six; ≥2 events at a dose level in 3–6 participants define that dose as exceeding the maximum tolerated dose (MTD), with the preceding dose as the recommended phase 2 dose.

Each participant undergoes a three‑day treatment phase followed by clinical and radiological follow‑up for up to one year. This design allows systematic dose escalation while ensuring patient safety and assessment of early efficacy signals.

### Participants and enrolment

Patients with recurrent glioblastoma who are referred for Gamma Knife treatment at Haukeland University Hospital, the national reference center for stereotactic radiosurgery in Norway, will be screened for eligibility. Those who meet all study criteria and provide informed consent will be enrolled and offered participation in the trial.

### Inclusion and exclusion criteria

The inclusion criteria for this study require that participants must be ≥18 years of age, have histologically confirmed glioblastoma (World Health Organization WHO grade 4) with a first or second recurrence as defined by Response Assessment in Neuro‑Oncology (RANO) criteria, and that they have previously undergone maximal safe resection followed by radiotherapy with concomitant temozolomide. Eligible patients must be suitable candidates for Gamma Knife stereotactic radiosurgery, with a recurrent lesion measuring 3 cm or less (≤ 15 cm³) on MRI obtained within the preceding 30 days prior to treatment. Functional status must be a Karnofsky Performance Status ≥70 and estimated life expectancy >12 weeks. To ensure adequate organ function, baseline laboratory values obtained within 7 days before first study dose must meet all of the following thresholds: absolute neutrophil count ≥1.0 × 10⁹/L without the support of filgrastim; platelet count ≥100 × 10⁹/L; hemoglobin ≥100 *g*/L; total bilirubin <1.5 × the upper limit of normal (ULN), except patients with Gilbert’s syndrome who may enroll if total bilirubin <51.3 µmol/L, alanine aminotransferase (ALT) and aspartate aminotransferase (AST) <3 × ULN; serum creatinine <1.5 × ULN, international normalized ratio (INR) <1.4. At least 4 weeks must have elapsed since any prior systemic anti‑cancer therapy, and all treatment‑related toxicities must have recovered to Grade 1 or lower before enrollment. All participants must provide written informed consent prior to any study‑specific procedures. .

Exclusion criteria apply to patients with known allergy or hypersensitivity to sulfasalazine, sulfonamides, salicylates, or related excipients, as well as those receiving or planning to receive temozolomide or other systemic anti‑tumor therapies during the study period. Individuals previously treated with sulfasalazine after their glioblastoma diagnosis, or those participating in a pharmacokinetic or immunotherapy trial within the past four weeks, will also be excluded. Additional exclusion criteria include medical conditions that could interfere with safe participation, such as glucose‑6‑phosphate dehydrogenase deficiency; significant cardiac (New York Heart Association functional classification III–IV), renal (chronic kidney disease (CKD) 3–5), or hepatic impairment; severe asthma or a history of severe allergic reactions; porphyria; erythema multiforme; intestinal or urinary obstruction; and the presence of another active malignancy or multiple sclerosis. Patients who are pregnant or breastfeeding, as well as those with psychiatric or cognitive disorders that impair the ability to provide informed consent or adhere to study procedures, will similarly be excluded.

#### Washout requirements for prior therapies at recurrence

Participants may have received one prior treatment for an earlier recurrence and may enroll at first or second recurrence, however, they must be bevacizumab‑naïve, as prior bevacizumab exposure may alter vascular permeability and confound safety assessments. A minimum of four weeks must have elapsed since any prior local or systemic anti‑cancer therapy, including alkylating agents such as temozolomide rechallenge, lomustine, or PCV, and all treatment‑related toxicities must have recovered to Grade ≤ 1 before study treatment.

Participants may have undergone resection or GKRS for a prior recurrence, but GKRS to the same previously treated lesion and prior fractionated reirradiation for recurrence are not permitted.

Prior exposure to investigational agents, including participation in pharmacokinetic or immunotherapy trials, or use of device‑based therapies such as tumor treating fields, is permitted only if ≥4 weeks have elapsed before enrollment and all toxicities have resolved to Grade ≤ 1. Tumor treating fields are not in routine use in Norway, but prior use does not preclude participation. No additional washout restrictions apply.

### Sample size

As is standard for phase I dose‑escalation studies using a 3 + 3 design, no formal sample size calculation was performed. The total number of participants is determined by the number of dose‑escalation cohorts required to identify the maximum tolerated dose. Based on the planned four dose levels, the study is expected to enroll between 12 and 24 patients, which is consistent with conventional 3 + 3 methodology and provides an adequate basis for assessing safety and tolerability in this early‑phase setting.

### Intervention

Participants are admitted to the hospital for five days, from Monday to Friday. Screening procedures and baseline assessments are conducted on the day of admission (Day 0). The intervention takes place over the subsequent three days (Day 1–3), and participants are discharged on Day 4 following an end‑of‑treatment safety evaluation.

During the intervention period, participants receive oral sulfasalazine in the form of enteric‑coated 500 mg tablets administered once daily for three consecutive days. Dose escalation follows a traditional 3 + 3 design, in which cohorts of three to six participants receive one of four predefined dose levels: 1.5 *g*, 3 *g*, 4.5 *g*, or 6 *g* once daily. All doses are administered under the supervision of a study nurse, who monitors intake and assesses acute tolerability.

On the third intervention day, approximately 6 hours after participants receive their third and final sulfasalazine dose, they undergo stereotactic gamma knife radiosurgery. GKRS is delivered with a standard prescription dose of 12 Gy to the contrast‑enhancing tumor volume.

### Justification of dose regimen

The selection of a once‑daily, 3‑day sulfasalazine regimen at dose levels from 1.5 to 6.0 *g* is based on pharmacokinetic considerations and on prior clinical experience, including the study by Robe et al [16], who used the same dose range in patients with malignant gliomas after primary treatment. Sulfasalazine is metabolized by intestinal bacteria to sulfapyridine and 5‑ASA. However, it is the parent compound and not its metabolites that inhibits xCT and provides the radiosensitizing effect. Most sulfasalazine undergoes colonic cleavage, with <15% reaching systemic circulation, and the serum half‑life is approximately 12.7 hours[18], although with substantial interindividual variability. With this pharmacokinetic profile, administering sulfasalazine over 3 days is expected to gradually increase intracellular drug exposure and reduce glutathione levels in tumor cells before GKRS.

The sulfasalazine dose required to achieve high occupancy of the xCT antiporter, as well as the turnover rate of sulfasalazine bound to xCT in patients, is not yet known. As the optimal biologically effective dose remains undefined, the present trial includes several dose levels in a standard dose‑escalation design to identify the highest dose considered safe. Robe et al. administered 1.5, 3.0, 4.5, and 6.0 *g* daily for a substantially longer duration (mean 32 days) as monotherapy to patients with recurrent malignant glioma (mean tumor volume 68 mL) until progression or treatment‑limiting toxicity. Although all patients experienced adverse effects including two fatalities there was no correlation between dose level and toxicity, and notably, six of seven grade 4 events and both deaths occurred in the lowest‑dose cohort (1.5 *g*). Importantly, in all cases where sulfasalazine was discontinued due to side effects, toxicity emerged far later than the brief 3‑day exposure used in our trial. Our study includes patients with considerably smaller tumor volumes (<15 mL) and uses stepwise dose escalation to ensure safety. In this context, employing the same dose range as in Robe et al. is considered feasible with acceptable risk. Moreover, these dose levels align with those used safely for decades in chronic inflammatory diseases such as rheumatoid arthritis and Crohn’s disease.

Although pharmacological data directly demonstrating sulfasalazine penetration of the intact blood–brain barrier are lacking, both the findings from Robe et al. and preclinical experiments detecting sulfasalazine in brain tumor tissue after 3 days of dosing, together with clear radiosensitizing anti‑tumor effects in glioblastoma xenograft models treated with GKRS, indicate that sulfasalazine reaches tumor tissue through the disrupted blood–brain barrier.

Once‑daily dosing is selected for feasibility and consistency with preclinical experiments and facilitates safe administration under direct nurse observation during inpatient hospitalization ensuring adherence. Although pill burden increases across dose levels (3 - 12 x 500 mg tablets), once‑daily administration is operationally simpler than divided dosing while maintaining alignment with the expected radiosensitization window. A single daily dose produces a well‑defined serum concentration peak 3 - 12 hours after ingestion, allowing predictable pharmacodynamic timing relative to GKRS.

#### Concomitant medications and supportive care

Chronic medication with sulfasalazine and ongoing treatment with temozolomide at study inclusion are exclusion criteria. All other prescribed medications may continue unchanged, including stable antiepileptic therapy. Dexamethasone is continued at the dose prescribed at admission with no required protocol‑mandated stabilization period. When clinically indicated due to asymptomatic or symptomatic peritumoral edema on baseline MRI, a pre‑procedural dose of dexamethasone (4 mg) may be administered before GKRS together with antacid prophylaxis such as ranitidine according to institutional practice.

All patients are offered premedication consisting of oxazepam 10 mg or diazepam 5 mg according to institutional practice prior to GKRS. Stereotactic frame placement is performed under local anesthesia with subcutaneous lidocaine with adrenaline (10 mg/ml up to 20 ml), with additional infiltration (up to 10 ml) as needed, which is the primary method of pain control. Additional anxiolysis or light sedation during GKRS is permitted when clinically appropriate.

Supportive care follows routine neurosurgical practice. Paracetamol, tramadol, or morphine is used as needed for headache or procedural discomfort when local anesthetic refill is insufficient, metoclopramide or ondansetron is administered for nausea and zopiclone is used for insomnia on the evening before or the day of GKRS. Over‑the‑counter medications, vitamins, herbal supplements, and other chronic treatments are allowed at the investigator’s discretion provided they do not interfere with study treatment or confound safety assessments. All concomitant medications are documented in the case report form, including a separate record of steroid use and any dose changes over time

#### Management of adverse events related to sulfasalazine and stopping rules

Sulfasalazine is administered once daily for only 3 days and has a plasma half‑life of 12.7 h therefore, drug‑related toxicity is expected to occur during or shortly after this brief exposure. Given the 24‑hour interval between a total of only three doses and scheduled daily laboratory monitoring, treatment holding is not feasible, only discontinuation criteria are applicable.

Gastrointestinal intolerance (nausea, vomiting, abdominal pain, diarrhea), hypersensitivity reactions (rash, fever, urticaria), hepatotoxicity (elevated bilirubin or transaminases) and hematologic toxicity (neutropenia, thrombocytopenia, anemia) of Grade ≤ 2 will be managed with symptomatic therapy (e.g., antiemetics or antihistamines) and close clinical and laboratory monitoring, with continued sulfasalazine treatment. Grade 3 neutropenia or thrombocytopenia will warrant discontinuation of sulfasalazine. Dosing may proceed only if the abnormality returns to Grade ≤1 within 24 h without intervention. Hematologic toxicities persisting for >24 h, any hematologic toxicity of Grade >3, and all Grade ≥3 gastrointestinal intolerance, hypersensitivity reactions, or hepatotoxicity will lead to permanent discontinuation of sulfasalazine and notification of the DMC

### Screening and baseline assessments

Screening is performed on Day 0 following pre‑screening at referral to confirm eligibility. All screening procedures, baseline imaging, laboratory tests, and functional assessments are completed prior to the first sulfasalazine dose.

Baseline imaging includes MRI and [¹¹C]methionine PET to verify recurrent glioblastoma, evaluated according to RANO criteria and assessment of metabolic tumor activity using the maximum standardized uptake value (SUV_max_). GSH-edited MRS is obtained to quantify baseline glutathione levels in both tumor tissue and normal brain.

Clinical assessments include detailed medical history, complete physical examination, and vital signs. Laboratory evaluations encompass hematologic, renal, hepatic, electrolyte, and inflammatory profiles, and pregnancy testing is performed when indicated. Functional status is assessed using KPS, and participants complete the Functional Assessment of Cancer Therapy-Brain (FACT‑Br) questionnaire to document baseline patient‑reported outcomes related to neurological function and QoL.

### Follow‑up assessments

#### Primary outcome

The dose‑limiting toxicity (DLT) window is defined as Day 1 (first dose of sulfasalazine) through 30 days after the final sulfasalazine dose and gamma knife treatment on Day 3 during which adverse events will be recorded continuously. Adverse events will be graded from 1 to 5 according to Common Terminology Criteria for Adverse Events (CTCAE) version 4.0 [19,20]. For each AE, investigators will assign attribution using standard categories (unrelated, unlikely, possibly, probably, or definitely) to determine the causal relationship to sulfasalazine and GKRS. DLT is defined as any CTCAE v4.0 grade ≥ 3 adverse event occurring within this window, assessed by the investigator as at least possibly related to trial intervention. Adverse events considered to be clearly attributable to expected disease progression or pre-planned hospitalization admission (ie, elective or scheduled surgery arranged prior to the start of treatment) will not be reported as DLT. Similarly, treatment‑related effects expected following GKRS (e.g. pseudoprogression or increased peritumoral edema) and events listed in the sulfasalazine SmPC are not considered “unexpected” and are only classified as Suspected Unexpected Serious Adverse Reactions (SUSAR) if more severe in intensity or more frequent than anticipated.

Safety monitoring includes daily clinical evaluations of vital signs and laboratory testing (hematology, renal/hepatic function, electrolytes, C‑reactive protein, and urine analysis) during the 5-day hospitalization (Day 0–4) in conjunction with the 3-day intervention period (Day 1–3) and at outpatient follow‑up visits at 1, 2, and 4 weeks. MRI will be performed on Day 4 and at 4 weeks to detect early edema and other acute treatment‑related changes. The 4‑week visit will also include [¹¹C]methionine PET. Participant‑reported outcomes during the safety monitoring period will be collected using the FACT‑Br questionnaire at Day 0, Day 4, and weeks 1, 2, and 4. KPS will be assessed at the same timepoints.

Dose escalation follows the 3 + 3 design and proceeds only if no participant experiences a treatment‑related CTCAE v4.0 ≥ 3 adverse event. If one participant experiences a grade ≥ 3 event, the cohort will be expanded to six participants. The maximum tolerated dose (MTD) is defined as the dose preceding that at which 2 of 3 or 2 of 6 patients experience a grade 3 or higher adverse event within the DLT-window.

To ensure adequate safety maturation, a minimum of 1 week must elapse after the completion of treatment on Day 3 in the preceding participant before the next participant is enrolled. Missed doses, dose reductions and early discontinuation will not remove a participant from DLT evaluability. Hospitalization does not remove a participant from DLT evaluability unless it is due to a pre‑planned elective procedure or expected disease progression rather than an adverse event related to sulfasalazine or GKRS. Serious adverse events (SAEs) will be reported according to regulatory timelines. Late radiation‑related toxicity will be monitored at 3, 6, 9 and 12 months post-GKRS, but will not influence dose‑escalation decisions.

#### DMC oversight

The independent Data Monitoring Committee (DMC) conducts a scheduled safety review 1 week after completion of treatment in the third participant of each cohort. This timing is considered adequate for capturing clinically relevant early grade ≥3 drug‑related adverse events as sulfasalazine is administered for only three days, has a plasma half‑life of approximately 12.7 h, and is known to cause early rather than delayed toxicity. However, any grade ≥3 adverse events occurring after the initial review within the DLT window will trigger an additional DMC assessment during which enrollment will be paused. Such events may result in cohort expansion or dose de‑escalation as appropriate.

In accordance with standard practice in phase I trials combining systemic agents with radiotherapy, late toxicities will not contribute to dose‑escalation decisions because they occur too long after investigational drug exposure to inform MTD determination. Nevertheless, clinically meaningful late radiation‑induced adverse effects, including those potentially modified by radiosensitization and typically emerging months to years after treatment, will be documented and monitored throughout longitudinal follow‑up

#### Secondary outcomes

Secondary outcome assessments will include clinical (KPS/FACT-Br) follow‑up and standard MRI (T1‑weighted with and without contrast, T2, and FLAIR) imaging at Day 4, 4 weeks, 12 weeks, and subsequently every three months for up to 12 months or until consent withdrawal, documented tumor progression, or death. Tumor response on MRI will be assessed according to RANO criteria for gliomas [21]. At the 1‑month visit, [¹¹C]methionine PET will be performed and evaluated according to PET Response Assessment in Neuro‑Oncology criteria (PET‑RANO), criteria [22] to differentiate pseudoprogression from true progression. GSH‑edited MRS to assess longitudinal changes in intratumoral and normal‑brain GSH levels will be obtained at Day 3, Day 4, and at 1 month.

#### Imaging methods

Baseline MRI, [¹¹C]methionine PET, and GSH‑edited MRS are performed at baseline before the first sulfasalazine dose. On Day 3, GSH‑edited MRS is acquired in the morning (approximately 09:00), after the third sulfasalazine dose (administered at approximately 07:00) but prior to GKRS (scheduled at around 12:00–14:00). This timing captures the pharmacodynamic effect of sulfasalazine immediately before irradiation. On Day 4, GSH‑edited MRS is repeated approximately 24 hours after GKRS to quantify post‑treatment changes in glutathione levels following three days of sulfasalazine exposure and single‑session radiosurgery. A full MRI ± [¹¹C]methionine PET ± GSH‑edited MRS assessment is performed at the 1‑month follow‑up visit to evaluate longer‑term metabolic and structural changes, including contrast enhancement, tumor size, and glutathione dynamics.

Subsequent MRI scans (T1 with and without contrast, T2, and FLAIR) are conducted at 3, 6, 9, and 12 months. PET imaging is repeated only when clinically indicated, such as in cases of suspected pseudoprogression.

GSH‑edited MRS is acquired on a 3T Siemens Biograph mMR system using a MEGA‑PRESS sequence with voxels located in the tumor (nominally 27 mL, with placement tailored to maximize coverage of the individual tumor) and healthy tissue (27 mL voxel in the midline anterior cingulate cortex (ACC), adjusted if necessary to avoid intersection with the tumor volume). Similar positioning will be maintained across visits for each individual. With tumor location and size varying between patients, the MRS voxel is placed judiciously on a per-individual basis to maximize intersection with (GD-enhancing) tumor regions, without extending into the ventricles or non-brain regions. Given the comparatively large volume needed for GSH measurement, voxels typically also overlap with smaller portions of the necrotic/non-enhancing core, peritumoral oedema and healthy tissue. Since the behavior of MRS-measured signals (e.g., decay rates for the metabolite and water reference signals) in the different regions is not well defined, there is currently no meaningful way to adjust for this numerically. Importantly, similar placement is maintained across return visits, so these «partial volume» effects are unlikely to substantially impact longitudinal analysis. Healthy reference voxels are placed medially in the ACC, with minor adjustments (slight shifts) in cases where the nominal placement would have intersected with the tumor volume. All MRS data are analyzed through a common pipeline (Gannet 3.3.2), with configuration adjusted (for the entire dataset) to accommodate factors associated with tumor spectra: most notably, spectral alignment is disabled, as this has been found to function poorly in the presence of extremely strong lipid signals and weak (or absent) signals from typical reference metabolites (NAA, Cr). This is outlined, along with quality control criteria and outcomes for the reconstructed spectra, in the MRSinMRS Table 1[23]^,^ [24].

**Table 1:** An MRSinMRS1 checklist for the Phase I Trial Combining Sulfasalazine and Gamma Knife Radiosurgery for Recurrent Glioblastoma. Protocol Identification Number: SAS-GKRS-TRIAL-01, EUDRACT Number: 2019–002,110-40.

Site (name or number)	Haukeland University Hospital
**1. Hardware**	
a. Field strength [T]	3 T
b. Manufacturer	Siemens
c. Model (software version if available)	Biograph mMR, VE11P
d. RF coils: nuclei (transmit/receive), number of channels, type, body part	mMR head/neck matrix coil (1H receive), 12/20 channels active
e. Additional hardware	N/A

**2. Acquisition**	
a. Pulse sequence	MEGA-PRESS (GSH editing, Universal implementation)
b. Volume of interest (VOI) locations	Subject-specific intra-tumoral and contralateral healthy tissue
c. Nominal VOI size [cm3, mm3]	Nominally 27 mL (30 × 30 × 30 mm^3^), some individual adaption
d. Repetition time (T_R_), echo time (T_E_) [ms, s]	T_R_ = 2000 ms, T_E_ = 80 ms
e. Total number of excitations or acquisitions per spectrumIn time series for kinetic studies i. Number of averaged spectra (NA) per time point ii. Averaging method (e.g. block-wise or moving average) iii. Total number of spectra (acquired/in time series)	256 transients total, alternating edit-ON/-OFF
f. Additional sequence parameters (spectral width in Hz, number of spectral points, frequency offsets) If STEAM:, mixing time (T_M_) If MRSI: 2D or 3D, FOV in all directions, matrix size, acceleration factors, sampling method	Spectral width 4000 Hz, 4096 data points 15 ms editing pulses at 4·56 ppm (edit-ON) and 7·5 ppm (edit-OFF)
g. Water suppression method	CHESS
h. Shimming method, reference peak, and thresholds for “acceptance of shim” chosen	3D-DESS (or FASTESTMAP)
i. Triggering or motion correction method (respiratory, peripheral, cardiac triggering, incl. device used and delays)	N/A

**3. Data analysis methods and outputs**	
a. Analysis software	Gannet 3.3.2
b. Processing steps deviating from quoted reference or product	Spectral alignment disabled to improve reliability in the presence of extremely strong lipid signal and potentially weak/absent major reference peaks
c. Output measure (e.g. absolute concentration, institutional units, ratio), processing steps deviating from quoted reference or product	Water-referenced estimates for GSH, *without* adjustment for voxel tissue content (traditional segmentation and assumed relaxation rates not suitable in tumor context)
d. Quantification references and assumptions, fitting model assumptions	N/A

**4. Data quality**	
a. Reported variables (SNR, linewidth (with reference peaks))	SNR GSH 8·05 [3·03,12·9] (healthy), 4·16 [2·.25,8·83] (lesion) SNR NAA 152 [99·7·247] (healthy), 77·4 [29·.3·153] (lesion) SNR Cr 130 [84·4·215] (healthy), 63·1 [20·9114] (lesion) FWHM GSH 10·1 [5·46,14·7] (healthy), 11·8 [5·15,23·11] (lesion) FWHM NAA 11·6 [8·66,21·67] (healthy) 18·1 [11·4,26·7] (lesion) FWHM Cr 10·2 [7·90,16·8] (healthy), 16·1 [10·2,22·8] (lesion) Reported as median [95% confidence interval]; FWHM in Hz
b. Data exclusion criteria	FWHM GSH > 30 Hz; SNR GSH < 2
c. Quality measures of postprocessing model fitting (e.g. CRLB, goodness of fit, SD of residual)	FitError GSH > 50%; any parameter estimate < 0 Extreme outliers (> 3 x median absolute deviation evaluated from baseline acquisitions (timepoint D1) on healthy side)

[¹¹C]methionine PET is performed on the integrated 3 T PET/MRI system [¹¹C]methionine is administered intravenously according to institutional weight‑based protocols, and imaging begins after a tightly controlled uptake time (10–20 min) to account for the short half‑life of carbon‑11 (20.3 min). PET data are reconstructed with MR‑based attenuation correction and standardized OASIS‑based processing. Volumes of interest (VOIs) are placed on co‑registered PET/MR images, with the lesion VOI centered on the region of highest methionine uptake while excluding necrotic or hemorrhagic tissue, and the reference VOI placed in contralateral normal‑appearing cortex or white matter. SUVmax and SUVmean are extracted, and tumor‑to‑normal (T/N) ratios are calculated using the contralateral reference region. All PET analyses are performed centrally by an experienced nuclear medicine physicist/radiologist using the prespecified OASIS pipeline to ensure reproducibility. PET‑RANO 1.0 criteria [25] are applied for metabolic response categorization.

#### Radiosurgery methods

GKRS is delivered in a single session using a Leksell stereotactic frame and contrast‑enhanced stereotactic MRI for target definition. The target is defined as the contrast‑enhancing tumor volume on T1‑weighted post‑contrast MRI with 1-mm slice thickness, with no additional margin. A prescription dose of 12 Gy is delivered to all lesions.

The planning goal is to use the 50% isodose line for optimal conformity and steep dose fall‑off. However, for very small lesions (≤ 1 cm³), a higher prescription isodose line (50–90%) may be selected to improve conformality and to minimize the 12‑Gy volume outside the target.

Plan quality is evaluated using standard GKRS metrics (tumor volume, prescription isodose, minimum and maximum dose, the 12‑Gy volume outside the target (V₁₂Gy), target coverage ratio, conformity index, selectivity index, and gradient index, dose rate) with planning objectives of a conformity ratio close to 1.0 (<1.05), selectivity >0.75, and a gradient index ≤3.0 (PCI <90%). These serve as guiding targets rather than strict constraints, with highest priority placed on achieving optimal target coverage while minimizing V₁₂Gy.

Normal‑tissue constraints follow institutional practice including brainstem V₁₂Gy < 1 ml, maximum dose to the optic apparatus < 8 Gy; and cochlear maximum dose < 4 Gy where applicable. Prior radiotherapy is incorporated into planning by restricting the treated volume to ≤ 15 cm³ and minimizing V₁₂Gy to normal brain. A fixed 12 Gy salvage dose is used independent of time since prior radiotherapy to minimize variability across cohorts. Patients previously treated with repeat fractionated reirradiation or GKRS for recurrence at the same target are excluded.

#### Justification for prescription dose

In the reirradiation setting, patients have already received 60 Gy to normal brain, creating a narrow therapeutic window. A 12 Gy prescription dose is consistent with our institutional practice and published experience. In a cohort of 77 rGBM patients treated with a median margin dose of 12 Gy (range 8–20 Gy) the median OS was 12 months, PFS 6 months, with <10% radiographic edema, and no treatment‑related neurological toxicity or radionecrosis. Importantly, outcomes did not improve with doses exceeding 12 Gy[8].

The 12 Gy isodose volume (V12Gy) is a recognized predictor of adverse radiation effects, and radionecrosis risk increases steeply as V12Gy expands. Even small increases in prescription dose or target volume can markedly increase toxicity without demonstrating a clear gain in OS. This is illustrated by Koga et al., where adding a 0.5 −1 cm margin to a 20 Gy plan did not improve OS but increased the rate of radionecrosis from 6.5% to 29%[26]. While Bunevicius et al. (n = 96) found that doses >15 Gy and treatment volumes ≤5 cm³ predicted longer OS in univariate analysis, only volume remained significant after multivariate adjustment, highlighting the dominance of the dose–volume relationship[27]. Several studies, however, illustrate the dose‑toxicity relationship as well. Imber et al. (n = 174, grade 4 glioma) reported a 13% incidence of radiation necrosis requiring surgery using a 16 Gy prescription dose, with a median OS of 10.6 months and prescription doses >15.5 Gy emerging as a positive predictor of OS[28]. In another study employing a 20 Gy prescription dose, the radionecrosis rate approached 40%, with 23% of patients requiring resection, while OS (11.3 months) remained similar to that seen in lower‑dose series[10]. Conversely, lowering the prescription dose below 12 Gy appeared to be safer, although not more effective, as reflected in the Vienna experience (median 10 Gy), which reported an OS of 9.6 months with no treatment‑related toxicity[29].

Although several retrospective studies have suggested that higher prescription doses (>14- 16 Gy) may be associated with improved PFS or OS, the OS in these trials seldom exceeds 12 months despite dose escalation. Consequently, dose–response relationships in the SRS literature must be interpreted with caution, as they are heavily confounded by tumor volume, tumor grade, and study heterogeneity. Most published reports are small, retrospective, single‑institution cohorts that mix WHO grade 3 and grade 4 gliomas, often lack IDH classification, combine SRS boost at diagnosis with SRS for recurrence, use different radiosurgical platforms and fractionation schemes, and apply prescription doses ranging from 10 to 20 Gy with variable target margins. Reported tumor volumes span from 0.01 to 99 cm³, further complicating assessment of any true dose–response relationship for GKRS in IDH‑wildtype recurrent glioblastoma following standard chemoradiation with the Stupp regimen. The ISRS guidelines recommend a prescription dose of 16 Gy, based on a meta analysis that combines WHO grade 3 and grade 4 gliomas[30]. Because grade 3 gliomas consistently demonstrate substantially longer survival than grade 4 gliomas, this tumor grade heterogeneity further obscures interpretation of dose–response relationships. For example, Dodoo et al. reported a median OS of 31.3 months for grade 3 versus 11.3 months for grade 4 at a 20 Gy prescription dose, with radionecrosis rates of 40%(10).

Taken together, our institutional data supports 12 Gy as a balanced single‑session GKRS dose with comparable survival rates as > 12 Gy studies while minimizing radionecrosis risk in previously irradiated, IDH‑wildtype GBM recurrence.

#### Response assessment

Tumor response versus treatment‑related change is assessed on longitudinal MRI per RANO 2.0[31], which requires a confirmation step to reduce misclassification of early post‑treatment effects. New or increasing enhancement on follow‑up is therefore classified as preliminary progression until a confirmatory MRI at 6–8 weeks determines confirmed PD or confirmed pseudoprogression, with the final status backdated to the preliminary timepoint. If MRI follow‑up remains inconclusive, amino‑acid PET may be used.

Pseudoprogression is defined as an early, transient treatment‑related imaging change that typically occurs within 12 weeks after radiation and stabilizes or improves without a change in therapy.

Radionecrosis is defined as a late, progressive radiation‑induced tissue injury occurring months to years after radiotherapy. Histopathology is the reference standard when resection is performed but is not required for diagnosis. All MRI timepoints are read centrally by an experienced academic neuroradiologist, independent of the treating team and blinded to the sulfasalazine dose level, ensuring consistent, unbiased interpretation across participants.

#### Endpoints

Because GKRS is a focal treatment, duration of response is evaluated using FFLP. FFLP is measured from GKRS to local progression of the trial‑treated lesion, with the date of MRI showing local failure as the event and participants without local failure censored at the last imaging follow‑up. PFS is measured from GKRS to any intracranial progression (either in the trial‑treated lesion or at distant intracranial sites) or death from any cause. The date of MRI demonstrating intracranial progression, or the date of death, constitutes the event; participants are censored at the last follow‑up if neither has occurred. OS is measured from GKRS to death from any cause. Death is the event, and survivors or participants lost to follow‑up are censored at the date of last known follow‑up. This structure ensures non‑overlapping endpoints. FFLP captures local failure only, with distant failure and death censored. PFS captures any intracranial progression or death, with participants alive without progression censored. OS captures death only, with survivors or those lost to follow‑up censored at their last known assessment.

### Data management and analysis

Study data will be recorded in protocol‑specific case report forms and monitored regularly for completeness, consistency, and accuracy. An independent DMC and steering committee will be appointed to oversee safety and the scientific integrity of the study and ensure adherence to the protocol, respectively.

All datasets will be de‑identified before analysis and stored in secure institutional repositories in accordance with local data‑protection regulations. Statistical analyses will be performed using validated software packages, SPSS and R.

The primary endpoint will be determination of the maximum tolerated dose and the recommended phase II dose of sulfasalazine administered as a radiosensitizer. The number of participants experiencing CTCAE v4.0. grade ≥ 3 adverse events will be summarized within each dose cohort, and formal hypothesis testing will not be required for dose determination.

Secondary endpoints will be summarized using descriptive statistics. Secondary endpoints will have limited statistical power due to small sample size and expected attrition related to disease progression or missed assessments. Time‑to‑event secondary outcomes (FFLP, PFS, OS) will be analyzed using Kaplan-Meier method and Cox models, while longitudinal secondary outcomes (tumor volume, KPS, FACT‑Br, SUVmax, and GSH-edited MRS-levels) will be analyzed using MLMs for repeated measures accommodating incomplete follow‑up under a missing at random assumption.

For tumor volume, KPS, and FACT‑Br, fixed effects will include time and interaction with time, with baseline score, age, and sex as covariates. Both intercept and slope of time will be included in the model as random effect factors. Prespecified pairwise contrasts across time points will use a Šidák adjustment for multiple comparisons. For GSH and SUVmax values, fixed effects will include region (tumor vs. normal‑appearing brain), time relative to baseline, and region by time interactions. .

### Method validation

This protocol addresses a critical unmet clinical need for patients with recurrent glioblastoma, a population with limited effective treatment options at the time of relapse. By targeting tumor redox homeostasis, the method aims to overcome a key mechanism of radioresistance and enhance the therapeutic effect of stereotactic radiosurgery. Preclinical studies demonstrate that modulation of glutathione‑dependent redox pathways can sensitize resistant tumors to radiation [32] and chemotherapy (33) by disrupting intrinsic antioxidant defense mechanisms. Sulfasalazine has been shown in preclinical models to inhibit this defense system by blocking glutathione synthesis, thereby increasing oxidative stress within tumor cells and creating a biologically grounded rationale for clinical evaluation.

The protocol is novel, and sulfasalazine has, to our knowledge, not previously been tested clinically as a radiosensitizer in combination with GKRS or any other radiotherapy modality. The study incorporates established and validated clinical techniques, including standard laboratory analyses, CTCAE v4.0. toxicity grading, KPS, FACT‑Br questionnaires, MRI, GSH‑edited MRS, and [¹¹C]methionine PET imaging. Dose‑escalation and safety monitoring follows the internationally recognized 3 + 3 design routinely used in phase I oncology studies.

Data generated during the study will establish feasibility and safety including the risk of late radiation‑induced effects. The trial will also provide mechanistic insight into treatment‑related changes in glutathione synthesis and tumor metabolism, as well as preliminary efficacy data regarding tumor response patterns and survival outcomes when combining sulfasalazine with GKRS.

In summary, this trial will generate the foundational data required to support a future phase II trial. If the strategy proves safe and feasible, repurposing an FDA‑approved, low‑cost oral drug as a short-course radiosensitizer could enable rapid progression to efficacy testing and may stimulate broader investigation of redox‑targeted radiosensitization across additional tumor types and radiation modalities.

## Limitations

This method has several limitations. As a phase I 3 + 3 dose‑escalation study, the sample size will be small, which limits the precision of safety estimates and reduces the ability to detect rare or delayed adverse events. Although the 3 + 3 design identifies the maximum tolerated dose, it provides limited information about dose response relationships and inter- patient variability. It is also possible that the therapeutic window may be wider than anticipated, such that lower doses could achieve meaningful radiosensitization even if they fall below the recommended dose determined by dose‑limiting toxicity criteria.

Radiation‑induced side effects usually emerge well beyond the traditional 30‑day reporting window used in phase I trials. The protocol partially compensates for this through scheduled MRI surveillance for late changes, documentation of radiation necrosis and surgical interventions when required, quality‑of‑life assessments, and [¹¹C]methionine PET imaging when clinically indicated.

The method relies on advanced neuro‑imaging modalities, including GSH‑edited MRS and [¹¹C]methionine PET, which require specialized equipment and expertise. This may limit reproducibility across centers lacking such resources. Early post‑treatment imaging may also be confounded by transient effects such as edema or pseudoprogression, complicating interpretation of metabolic and structural changes and savage treatments for new lesions may further confound radiological and clinical outcome assessments.

Because this is a single‑arm study, efficacy outcomes such as PFS and OS cannot be directly compared with a control group. Historical data for recurrent isocitrate dehydrogenase (IDH)‑wildtype glioblastoma treated with SRS after completion of the Stupp protocol are sparse or lacking, limiting outcome comparisons with prior studies. Glioblastoma is a diffuse disease, whereas GKRS is a focal treatment. Although selective enhancement of the GKRS-effect within the contrast enhancing recurrence through redox targeting may amplify a local inflammatory and hence antitumor directed immune response, it is unlikely to produce a systemic abscopal effect capable of altering the natural history of distant recurrences. The protocol does not include trial treatment for subsequent GKRS-treatments for new recurrences arising outside the treated volume. Finally, strict inclusion criteria essential for safety may reduce generalizability, meaning that study participants may not fully represent the broader population of patients receiving SRS for glioblastoma recurrence.

## Ethical statements

The study will be conducted in accordance with the Declaration of Helsinki, International Council for Harmonisation – Good Clinical Practice (ICH‑GCP) guidelines, and applicable national regulatory requirements. The full study protocol, patient information sheet, and informed consent form will be reviewed and approved by the Regional Ethics Committee and the national Competent Authority prior to enrolment. The trial will be prospectively registered on ClinicalTrials.gov before inclusion of the first participant. Written informed consent will be obtained from all participants after they have received comprehensive verbal and written information about the study procedures, potential risks, and their rights. Participation will be voluntary, and withdrawal will not affect further clinical care. All personal data will be handled in accordance with national data‑protection legislation, and confidentiality will be ensured throughout data collection, analysis, reporting, and publication. Participants will be identified only by study ID and initials, and no identifiable information will be shared with third parties

## Declaration of competing interest

The authors declare that they have no known competing financial interests or personal relationships that could have appeared to influence the work reported in this paper.

## Data Availability

No data was used for the research described in the article.

## References

[1] Ostrom Q.T., Cioffi G., Gittleman H., Patil N., Waite K., Kruchko C. (2019). CBTRUS statistical Report: primary brain and other Central nervous system tumors diagnosed in the United States in 2012-2016. Neuro Oncol..

[2] Stupp R., Mason W.P., van den Bent M.J., Weller M., Fisher B., Taphoorn M.J. (2005). Radiotherapy plus concomitant and adjuvant temozolomide for glioblastoma. N. Engl. J. Med..

[3] Weller M., Tabatabai G., Kastner B., Felsberg J., Steinbach J.P., Wick A. (2015). MGMT promoter methylation is a strong prognostic biomarker for benefit from dose-intensified temozolomide rechallenge in progressive glioblastoma: the DIRECTOR trial. Clin. Cancer Res..

[4] Fu X., Shi D., Feng Y. (2022). The efficacy and safety of adjuvant lomustine to chemotherapy for recurrent glioblastoma: a meta-analysis of randomized controlled studies. Clin. Neuropharmacol..

[5] Taal W., Oosterkamp H.M., Walenkamp A.M., Dubbink H.J., Beerepoot L.V., Hanse M.C. (2014). Single-agent bevacizumab or lomustine versus a combination of bevacizumab plus lomustine in patients with recurrent glioblastoma (BELOB trial): a randomised controlled phase 2 trial. Lancet Oncol..

[6] Stupp R., Wong E.T., Kanner A.A., Steinberg D., Engelhard H., Heidecke V. (2012). NovoTTF-100A versus physician's choice chemotherapy in recurrent glioblastoma: a randomised phase III trial of a novel treatment modality. Eur. J. Cancer.

[7] Walker M.D., Strike T.A., Sheline G.E (1979). An analysis of dose-effect relationship in the radiotherapy of malignant gliomas. Int. J. Radiat. Oncol. Biol. Phys..

[8] Skeie B.S., Enger P.O., Brogger J., Ganz J.C., Thorsen F., Heggdal J.I. (2012). gamma knife surgery versus reoperation for recurrent glioblastoma multiforme. World Neurosurg..

[9] Blakstad H., Brekke J., Rahman M.A., Arnesen V.S., Miletic H., Brandal P. (2023). Survival in a consecutive series of 467 glioblastoma patients: association with prognostic factors and treatment at recurrence at two independent institutions. PLoS. One.

[10] Dodoo E., Huffmann B., Peredo I., Grinaker H., Sinclair G., Machinis T. (2014). Increased survival using delayed gamma knife radiosurgery for recurrent high-grade glioma: a feasibility study. World Neurosurg.

[11] Lewerenz J., Hewett S.J., Huang Y., Lambros M., Gout P.W., Kalivas P.W. (2013). The cystine/glutamate antiporter system x(c)(-) in health and disease: from molecular mechanisms to novel therapeutic opportunities. Antioxid. Redox. Signal..

[12] Sontheimer H., Bridges R.J (2012). Sulfasalazine for brain cancer fits. Expert. Opin. Investig. Drugs.

[13] Human Protein Atlas. SLC7A11 expression summary [Internet]. Available from: https://www.proteinatlas.org/ENSG00000151012-SLC7A11/pathology.

[14] Sleire L., Skeie B.S., Netland I.A., Forde H.E., Dodoo E., Selheim F. (2015). Drug repurposing: sulfasalazine sensitizes gliomas to gamma knife radiosurgery by blocking cystine uptake through system Xc-, leading to glutathione depletion. Oncogene.

[15] Takeuchi S., Wada K., Nagatani K., Otani N., Osada H., Nawashiro H. (2014). Sulfasalazine and temozolomide with radiation therapy for newly diagnosed glioblastoma. Neurol. India.

[16] Robe P.A., Martin D.H., Nguyen-Khac M.T., Artesi M., Deprez M., Albert A. (2009). Early termination of ISRCTN45828668, a phase 1/2 prospective, randomized study of sulfasalazine for the treatment of progressing malignant gliomas in adults. BMC. Cancer.

[17] Le Tourneau C., Lee J.J., Siu L.L (2009). Dose escalation methods in phase I cancer clinical trials. J. Natl. Cancer Inst.

[18] Kadife E., Decloedt E., Louw V., Abdulla Z.B., Kellermann T., Pillay-Fuentes Lorente V. (2026). Safety and pharmacokinetics of sulfasalazine and its metabolite sulfapyridine for treatment of preterm preeclampsia in Australia (SIP): an early phase, unblinded, single-arm, proof of concept clinical trial. EClinicalMedicine.

[19] Institute NC (2009).

[20] Wen P.Y., van den Bent M., Youssef G., Cloughesy T.F., Ellingson B.M., Weller M. (2023). RANO 2.0: update to the response assessment in neuro-oncology criteria for high- and low-grade gliomas in adults. J. Clin. Oncol..

[21] Albert N.L., Preusser M. (2024). Measure what is measurable: PET RANO 1.0 criteria for interpretation of amino acid PET of diffuse gliomas. Neuro Oncol..

[22] Lin A., Andronesi O., Bogner W., Choi I.Y., Coello E., Cudalbu C. (2021). Minimum reporting Standards for in vivo Magnetic resonance spectroscopy (MRSinMRS): experts' consensus recommendations. NMR Biomed..

[23] Saleh M.G., Rimbault D., Mikkelsen M., Oeltzschner G., Wang A.M., Jiang D. (2019). Multi-vendor standardized sequence for edited magnetic resonance spectroscopy. Neuroimage.

[24] Albert N.L., Galldiks N., Ellingson B.M., van den Bent M.J., Chang S.M., Cicone F. (2024). PET-based response assessment criteria for diffuse gliomas (PET RANO 1.0): a report of the RANO group. Lancet Oncol..

[25] Koga T., Maruyama K., Tanaka M., Ino Y., Saito N., Nakagawa K. (2012). Extended field stereotactic radiosurgery for recurrent glioblastoma. Cancer.

[26] Bunevicius A., Pikis S., Kondziolka D., Patel D.N., Bernstein K., Sulman E.P. (2022). Stereotactic radiosurgery for glioblastoma considering tumor genetic profiles: an international multicenter study. J. Neurosurg..

[27] Imber B.S., Kanungo I., Braunstein S., Barani I.J., Fogh S.E., Nakamura J.L. (2017). Indications and efficacy of Gamma knife stereotactic radiosurgery for recurrent glioblastoma: 2 decades of institutional experience. Neurosurgery..

[28] Frischer J.M., Marosi C., Woehrer A., Hainfellner J.A., Dieckmann K.U., Eiter H. (2016). Gamma knife radiosurgery in recurrent glioblastoma. Stereotact. Funct. Neurosurg..

[29] Pinzi V., Kotecha R., Sahgal A., Gorgulho A., Jane Lim-Fat M., Levivier M. (2025). International Stereotactic Radiosurgery Society (ISRS) practice guidelines for Radiosurgery in recurrent high-grade glioma. Neuro Oncol..

[30] Ellingson B.M., Sanvito F., Cloughesy T.F., Huang R.Y., Villanueva-Meyer J.E., Pope W.B. (2024). A neuroradiologist's guide to operationalizing the response assessment in neuro-oncology (RANO) criteria version 2.0 for gliomas in adults. AJNR Am. J. Neuroradiol..

[31] Leung J.K., Panchal R., Anbalagan S., Durie E.P., Mansfeld J., Somaiah N. (2025). Modulating redox biology to improve radiation responses. Cancer J..

[32] Li Y., Zhang X., Wang Z., Li B., Zhu H. (2023). Modulation of redox homeostasis: a strategy to overcome cancer drug resistance. Front. Pharmacol..

